# “The emerging role of lymphocytes in post-stroke inflammation: A treatable target and review of current pharmacological evidence in humans"

**DOI:** 10.1016/j.bbih.2026.101172

**Published:** 2026-01-08

**Authors:** A. Papageorgiou, D. Chatzistefanidis, M. Nikolakea, N.-R. Karela, M. Gkrampovari, K. Lantavos, D. Bartzi, L. Traikov, S. Markoula

**Affiliations:** a University of Ioannina, Medical School, Department of Neurology, Greece; b Medical University-Sofia, Department of Medical Physics and Biophysics, Bulgaria; c National and Kapodistrian University of Athens, Medical School, Greece

**Keywords:** Stroke, Inflammation, Lymphocytes, Pharmacological approaches

## Abstract

Acute ischemic stroke is one of the leading causes of mortality and morbidity worldwide. The underlying inflammation process following the ischemic event arises as an important factor of great therapeutic interest. Recent research has showcased the roles of B- and T-lymphocytes in the acute post-stroke period, with various types of lymphocytes affecting differently the clinical outcome of the patient. Herein, we reviewed the literature and discussed the functional role of various subpopulations of lymphocytes in recovery and repair of the ischemic tissue as well as their influence on the final outcome of the patient. Additionally, we searched the literature regarding current knowledge on various drugs possibly affecting neuroinflammation or exhibiting a neuroprotective role in the acute post-stroke period.

## 1 Introduction

Stroke remains the second leading cause of mortality and morbidity worldwide. Acute ischemic stroke (AIS) accounts for approximately 70 % of all acute cerebrovascular events. The underlying pathophysiology of AIS is linked to chronic cerebrovascular changes strongly related to undergoing inflammatory processes. In addition, it has been shown that the inflammatory response triggered directly after the acute ischemic event may significantly affect patient mortality and overall prognosis. Inflammation emerges therefore as a promising therapeutic target in stroke management, as well as source of possible biomarkers for predicting clinical outcomes and overall long-term prognosis (Tadi and Lui, 2025) (see Table 1, Table 2, Table 3).

**Table 1:** The role of different B-lymphocytes in ischemic Stroke recovery.

B-Cell Subtype/Status	Key Actions	Downstream Effect	Clinical Impact	References
**↓ IL-10 –Producing B-Cells**	- Reduced anti-inflammatory signaling - Increased accumulation of monocytes and T-cells	- Enhanced neuroinflammation	- Larger infarct volume - Worse outcomes	(Seifert et al., 2018; Gesuete et al., 2016a, 2016b; Wang et al., 2023b)
**↑ IgA** **^+^** **B-Cells**	- Promotion of inflammatory cell infiltration (monocytes, T-cells)	- Sustained neuroinflammation	- Increased infarct size – Cognitive impairment - Impaired clinical recovery	(Zbesko et al., 2021; Doyle et al., 2015)
**Total B-Lymphocyte Activity**	- Immunosuppressive functions - Reduction in infarct volume - Enhancement of oxygen and blood supply	- Tissue protection and immune regulation	- Accelerated ischemic tissue repair - Improved functional outcome	(Wu et al., 2024a; Seifert et al., 2018; Gesuete et al., 2016a, 2016b; Wang et al., 2023b; Zbesko et al., 2021; Doyle et al., 2015)

**Table 2:** The role of different T-lymphocytes in ischemic Stroke recovery.

T-Cell Subtype	Key Actions	Downstream Effects	Clinical Impact	References
**Th1 Cells**	- Production of reactive oxygen species (ROS) - Secretion of interferon-γ (IFN-γ) - Release of chemokines	- Disruption of the blood–brain barrier (BBB)	- Worsened stroke outcome	Malone et al. (2019)
**Th2 Cells**	- Secretion of anti-inflammatory cytokines (IL-4, IL-5, IL-10, IL-13) - Production of nerve growth factor (NGF)	- Anti-inflammatory response - Tissue repair and angiogenesis - Debris clearance	- Improved functional recovery - Reduced post-stroke infections	(Cao et al., 2023), (Arumugam et al., 2005)
**T-regulatory Cells (Tregs)**	- Suppression of inflammation - NGF and amphiregulin production - Induction of oligodendrocytogenesis - White matter repair	- Microvascular restoration - BBB protection	- Neuroprotection - Early functional recovery - Protection against ischemic damage	(Ito et al., 2019a; Liesz et al., 2015; Benakis et al., 2022)
**Th17, CD4^+^Th1, CD8^+^T Cells**	- Secretion of pro-inflammatory cytokines - Amplification of inflammatory and thrombotic responses	- BBB disruption - Neutrophil recruitment - Increased infarct size	- Exacerbated brain injury - Worsened neurological deficits	(Nowik et al., 2007; Tuttolomondo et al., 2015; Masztalewicz et al., 2013; Na et al., 2015)

**Table 3:** Therapeutic agents in ischemic stroke: Phases, actions, and benefits.

Drug/Substance	Stage of Ischemic Stroke	Mechanism of Action/Benefits
**Heparin**	Acute phase	Anticoagulant; reduces morbidity and mortality without significant bleeding risk (Chamorro et al., 2000; Levi et al., 2007; Levy et al., 2009)
**Aspirin + Dipyridamole**	Early phase	Antiplatelet; reduces inflammation and improves clinical outcome (Worthmann et al., 2012a)
**Triflusal**	Early phase	Reduces IL-6, MIP-1, MCP-1; limits necrotic damage (Alvarez-Sabín et al., 2009)
**Statins (Pravastatin, Atorvastatin)**	Early to late phases	Reduces CRP, IL-6, TNF-α; anti-inflammatory and neuroprotective (Montaner et al., 2008a)
**Statins + Ezetimibe**	Early to late phases	Reduces inflammatory area, thrombin activity, and atherosclerosis (van Kuilenburg et al., 2011a; Kitagawa et al., 2017a; Yu et al., 2017a)
**Paracetamol**	Acute phase	Reduces fever; improves functional outcome (de Ridder et al., 2015a; Ridker et al., 1997a)
**Vinpocetine**	Post-acute/recovery	B-cell modulation; improves neural recovery (Zhang et al., 2018)
**Flogenism (Bromelain, Trypsin, Rutin)**	Maintenance/recovery	Anti-inflammatory, fibrinolytic, and antithrombotic effects (Vinychuk and Cheren'ko, 2007)
**Interleukin-10**	All phases	Anti-inflammatory cytokine; improves stroke and hemorrhage outcomes (Pelidou et al., 1999)
**Anti-inflammatory + antioxidant agents** (e.g., Mitoquinone, Edaravone, Ebselen, Lubeluzole)	Post-acute phase	Reduce reactive oxygen species (ROS); protect mitochondria; improve outcomes (Bi and Song, 2011; Shirley et al., 2014)
**Fingolimod**	Acute phase (with alteplase)	Immunomodulator; enhances recanalization, reduces reperfusion injury (Raju et al., 2012a, 2012b)**,** (Zhang et al., 2017; Zhu et al., 2015; Bo et al., 2024)
**Dimethyl Fumarate (DMF)**	Post-acute	Reduces neutrophils, macrophages, dendritic cells; neuroprotective and antioxidant (Owjfard et al., 2023; Zhang et al., 2024)
**Interferon Beta-1a**	Acute/subacute	Reduces proinflammatory cytokines; preserves BBB integrity (Kuo et al., 2016; Wanve et al., 2019)
**Monoclonal antibodies** (e.g., Enlimomab, Natalizumab)	Acute phase	No benefit; potential worsening of outcome (Enlimomab Acute Stroke Trial Investigators, 2001; Simats et al., 2016; Elkind et al., 2020a)
**DL-3-n-butylphthalide (NBP)**	Acute to subacute	Neuroprotective; improves 90-day functional status (Lv et al., 2022)
**Mildronate**	Acute phase	Anti-ischemic; restores cerebral blood flow (Zhu et al., 2013)
**Sodium Valproate**	Maintenance	HDAC9 inhibition; protects synapses; reduces recurrent stroke risk (Brookes et al., 2018)
**RAS inhibitors (ACEIs, ARBs)**	All phases	Anti-thrombotic and anti-inflammatory; platelet modulation (Chae et al., 2014)
**GLP-1 Receptor Agonists (GLP-1 RAs)**	All phases	Reduces infarct volume; promotes neurogenesis and CBF improvement (Goldenberg et al., 2022)
**Thiazolidinediones (e.g., Pioglitazone)**	Post-acute/maintenance	Anti-inflammatory; protects against neural degeneration (Culman et al., 2012)
**Vitamin B-complex and C**	Maintenance	Reduces oxidative stress and inflammatory factors (CRP, MCP-1, homocysteine); stabilizes atherosclerotic plaque (Sánchez-Moreno et al., 2004; Ullegaddi et al., 2006; Nenseter et al., 2009; Potter et al., 2009)
**BNG-1 (Chinese herbal compound)**	Acute/under research	Potential anti-thrombotic effect similar to aspirin (Chang et al., 2015)
**Licorice**	Acute/under research	May improve neurological outcomes (Ravanfar et al.)
**Ginsenoside Rd**	Acute/recovery	Anti-inflammatory; suppresses microglial proteasome activity; fewer side effects than glucocorticoids (Zhang et al., 2016)
**Ginkgolide, Melatonin**	Acute to recovery	Neuroprotective; associated with better NIHSS scores and clinical outcomes (Dong et al., 2020; Mehrpooya et al., 2022)
**Acupuncture**	Recovery/adjunct therapy	May reduce inflammation and improve neurological function (Tsai et al., 2024)

The immediate post-stroke inflammatory response begins with the disruption of blood-brain barrier (BBB), allowing circulating leukocytes to infiltrate the ischemic brain tissue. This is followed by an activation of macrophages and microglia residing in the ischemic area, while astrocytes begin to proliferate. These cellular and inflammatory responses contribute to increased loss of neuronal cells and demyelinization (Podjaski et al., 2015). Moreover, reperfusion injury resulting from restoration of blood flow to the occluded vessel may lead to an exacerbation of the inflammatory response through further disruption of the cerebrovascular autoregulation and BBB. This can subsequently lead to enhanced infiltration of white blood cells into the ischemic tissue (Mizuma et al., 2017; Aloizou et al., 2021).

Among the various pathophysiologic pathways involved in post-stroke inflammation, the role of lymphocytes may be pivotal in determining patient outcome. Activation of T-cells in acute ischemic lesions is strongly regulated by CD8^+^ cytotoxic T- and CD4^+^ Th1-cells, which contribute to brain tissue damage through the release of proinflammatory cytokines such as interferon-gamma (IFN-γ) and interleukin-17 (IL-17) (Yilmaz et al., 2006).

On the other hand, CD4^+^ Th2-cells, which exhibit anti-inflammatory properties, play a comparatively less dominant role; however, their cytokine profile may act beneficially to the damaged tissue (Wang et al., 2023a). Shortly after the ischemic event, the peripheral immune system becomes highly activated. Animal studies suggest that a systemic immune response of Th2-cells contributes to neuroprotection and axonal regeneration following acute brain and spinal cord injuries (Santamaría-Cadavid et al., 2020)**.** Nonetheless, this anti-inflammatory Th2 response may also contribute to stroke-induced immunodeficiency, which compromises cell-mediated immunity and increases susceptibility to infections (Miller and Elkind, 2016)**.**

Similarly, regulatory T-cells (Treg) produce anti-inflammatory cytokines such as interleukin-10 (IL-10) and transforming growth factor-beta (TGF-β), thereby limiting neuroinflammation and promoting tissue repair through neuroprotective mechanisms (Wang et al., 2022). Several studies suggest that Treg cells may be able to limit growth of the infarct volume by various mechanisms, including the secretion of TGF-β (Wang et al., 2021), IL-35 (Stanzione et al., 2022)**,** IL-10 (Liesz et al., 2009) or through their broader immunosuppressive effects (Zhang et al., 2021a)**.**

Given these findings, neuroinflammation emerges as a promising therapeutic target to improve long-term outcomes in stroke survivors. Various substances have been tested in experimental models and clinical studies, yielding mixed results. It remains to be determined how immunomodulatory or immunosuppressive therapies can be used effectively while minimizing potential safety concerns.

This review aims to provide a comprehensive overview of the current understanding of the role of B and T lymphocytes in the immediate post-stroke period. Additionally, we review the existing literature to highlight evidence supporting the potential benefits of various treatments in human subjects. These findings may guide future research into novel therapeutic agents showing promise in post-stroke recovery.

## 2 Methods

**Fig. 1:**
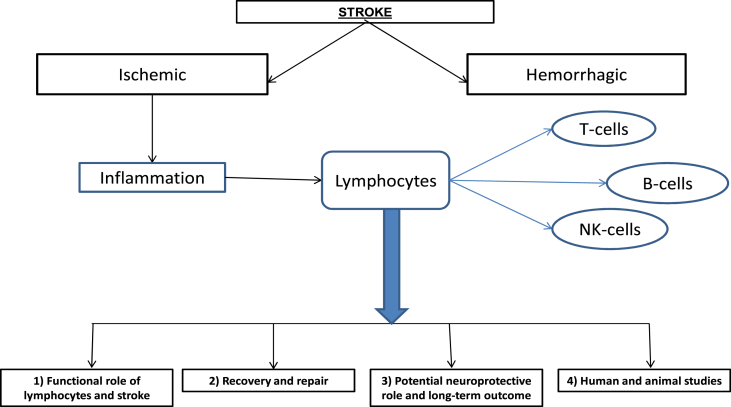
The relationship between lymphocytes and iscemic stroke.

The primary focus of this study is to review the existing literature on the role of B and T lymphocytes in the immediate post-ischemic recovery phase and their impact on the clinical outcome and overall prognosis of patients with ischemic stroke, as illustrated in Fig. 1. Additionally, we examine current evidence on therapeutic interventions involving these lymphocytes in both human and animal studies, with the aim of improving functional outcomes following an ischemic stroke.

We conducted a comprehensive literature search using PubMed, Scopus, and the World Health Organization (WHO) databases to identify studies examining the role of lymphocytes during the acute phase of ischemic stroke. Search terms included combinations of medical keywords such as “ischemic stroke and lymphocytes,” “role of lymphocytes in ischemic stroke,” “T-cells and ischemic stroke,” “B-lymphocytes and ischemic stroke,” “natural killer cells and ischemic stroke,” and “inflammatory process during stroke.” Additionally, we manually screened the reference lists of relevant articles to identify further studies that met our inclusion criteria.

We also reviewed the literature on pharmacological interventions evaluated during the acute phase of ischemic stroke in human subjects. Search terms included combinations such as “early therapy after ischemic stroke,” “ischemic stroke and inflammation,” and “ischemic stroke and medical therapy.” Furthermore, we explored the term “ischemic stroke” in combination with specific therapeutic agents, including heparin, aspirin, statins, and drugs used in the treatment of multiple sclerosis, such as fingolimod, dimethyl fumarate, interferons, and monoclonal antibodies.

To identify studies relevant to therapeutic strategies in ischemic stroke, we screened all abstracts and included only those investigating potential treatments targeting inflammatory responses during the immediate post-stroke period in human subjects. Case reports and articles written in languages other than English were excluded. Reference lists of the included studies were also examined to identify any additional relevant publications not captured in the initial database search.

## 3 Results

Our search yielded a total of 116 articles related to the role of lymphocytes in ischemic stroke. After screening the abstracts, 50 articles were excluded due to irrelevance to the scope of our study. The remaining 66 articles were included for full-text analysis and were further categorized based on their focus on the function of B or T lymphocytes, including their respective subpopulations. These articles were also classified according to the specific functional roles of lymphocytes during the acute phase of ischemic stroke, their involvement in the recovery and repair processes, their potential neuroprotective effects, and their impact on long-term patient outcomes. Both human and animal studies were examined to assess the positive or negative associations between lymphocytes and ischemic stroke pathophysiology.

### 3.1 Functions and mechanism of action of lymphocytes in ischemic stroke

Inflammation plays a crucial role in the clinical progression of ischemic stroke (Huang et al., 2006)**.** Lymphocytes are key contributors to this inflammatory response due to their abundant infiltration into ischemic brain tissue, where they exert both beneficial and detrimental effects (Zhang et al., 2021b)**.** This inflammatory response, triggered by internal or external factors, promotes the recruitment and accumulation of various lymphocyte subsets from peripheral lymphoid organs and lymph nodes into the brain (Sakai and Kobayashi, 2015). The impact of lymphocytes during the acute post-stroke phase largely depends on their specific subtype. Furthermore, the neutrophil-to-lymphocyte ratio (NLR) serves as an important prognostic marker; an elevated or stable neutrophil count accompanied by lymphopenia has been showed to be associated with poorer clinical outcomes (Juli et al., 2021).

#### 3.1.1 B-cells

B-cells appear to have a complex but overall beneficial role in the clinical outcome of ischemic stroke. Among them, regulatory B-cells (B-regs) have shown immunoregulatory properties that reduce central nervous system (CNS) damage following acute ischemic stroke. During the acute phase, B-reg cells suppress the activation of proinflammatory cells such as T-cells, monocytes, and microglia, leading to improved stroke outcomes (Bodhankar et al., 2013a). The absence of IL-10–producing B-regs correlates with larger infarct volumes and increased infiltration of inflammatory cells, including monocytes and T-cells (Engler-Chiurazzi et al., 2020).

In the recovery and repair phases, B-lymphocytes may play a pivotal role by decreasing infarct size and facilitating restoration of blood flow and oxygen supply. Although the current data remain limited and somewhat controversial, emerging evidence supports the potential of B-cells to promote functional recovery of injured brain tissue (Wu et al., 2024a). Both human and animal studies suggest that B-regs exert anti-inflammatory effects partly by recruiting regulatory T-cells (T-regs), which themselves are increasingly recognized for their neuroprotective functions in stroke, as discussed later.

Activation of B-regs has been associated with significant reductions in ischemic tissue volume, correlating with improved clinical outcomes and better response to treatment. Notably, recent murine studies highlight the potent neuroprotective effects of IL-10–producing CD5^+^ and CD1d^hi^ B-reg which mitigate infarct size and suppress maladaptive inflammation by inhibiting activation of peripheral immune cells and their migration into the brain (Seifert et al., 2018)**.**

Furthermore, the therapeutic potential of B-regs has also led to the identification of novel biomarkers for prognosis. For example, IL-10 expression in the affected hemisphere is being explored as a predictive marker in experimental stroke models. Mice depleted of B-cells demonstrate increased stroke severity, with larger infarcts, higher morbidity, and mortality compared to controls with intact B-cell populations, which show milder complications and improved outcomes (Seifert et al., 2018)**.**

The neuroprotective effects of B-cells may stem from their ability to modulate the inflammatory milieu post-stroke. Ischemic stroke triggers pro-inflammatory reactions that recruit peripheral immune cells to the injury site. Regulatory immune cells, including B-cells, respond by producing neuroprotective mediators. For instance, ischemic preconditioning—a process involving exposure to a mild ischemic insult—results in B-cell accumulation and confers protection against subsequent stroke events (Gesuete et al., 2016a)**.** Neuroprotection in immediate post-stroke period is mainly a function of B-cells and T-reg cells as discussed later. B-cell dysfunction is linked to poorer post-stroke prognosis, especially in the presence of cardiovascular comorbidities such as diabetes mellitus (Gesuete et al., 2016b). Through immunosuppression, B-cells facilitate earlier repair and recovery of ischemic brain tissue (Seifert et al., 2018).

**Fig. 2:**
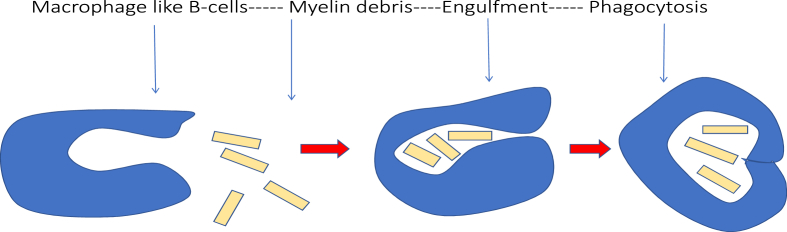
The process of phagocytosis and debris removal through macrophage B-cells.

In addition, a distinct subpopulation of B-cells, referred to as macrophage-like B-cells, has been shown to activate gene programs and molecular pathways that rapidly initiate phagocytosis. Their phagocytic activity, accompanied by the subsequent upregulation of phagosome- and lysosome-associated genes, facilitates the efficient removal of myelin debris following cerebral ischemia. Through this mechanism, neuronal recovery is accelerated, with reduced structural damage and preservation of neural signal integrity. The phagocytic capacity of this B-cell subset appears to modulate the post-ischemic immune response, thereby limiting the spread of inflammation to adjacent brain regions, as illustrated in Fig. 2 (Wang et al., 2023b).

Conversely, certain B-cell subsets may contribute negatively to stroke outcomes. Specifically, IgA^+^ B-cells have been implicated in cognitive decline and vascular dementia following stroke. Although mechanisms remain incompletely understood, immunohistochemistry and flow cytometry analyses reveal that IgA^+^ B-cells independently accumulate in damaged brain regions. High levels of IgA antibodies, potentially arising from somatic hypermutation of immunoglobulin genes, correlate with worsened cognitive and functional outcomes (Zbesko et al., 2021)**.** Furthermore, IgA + B-lymphocytes seem to be related to development of mental dysfunction, resulting in late onset cognitive deficits and eventually dementia. Supporting this, experimental models show that various antibody-producing lymphocytes—including IgM, IgG, and IgA—invade infarcted tissue and promote cognitive deficits. Postmortem studies of human stroke patients with dementia similarly demonstrate lymphocyte activation within affected brain areas (Doyle et al., 2015)**.**

#### 3.1.2 Τ-cells

**Fig. 3:**
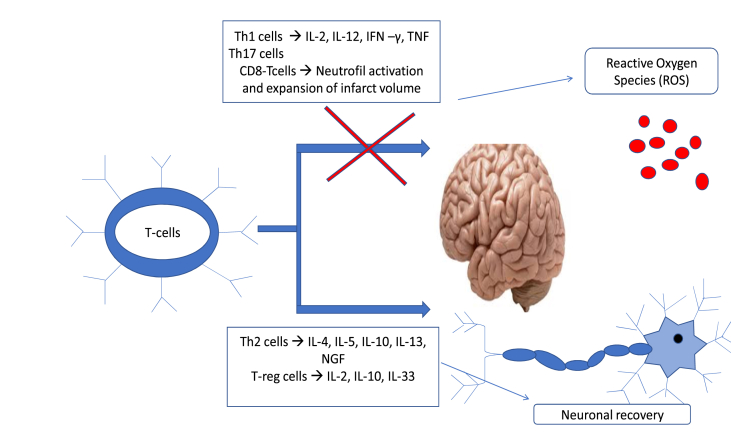
The function of different groups of T-lymphocytes in nervous system recovery after Stroke.

Brain inflammation is strongly regulated by T-cells, which can exert both protective and detrimental effects depending on the ischemic milieu. Initial observations suggested that T-cell deficiency was neuroprotective, leading to therapeutic strategies aimed at reducing T-cell populations to mitigate post-ischemic inflammation. However, these approaches paradoxically resulted in increased susceptibility to pathogenic infections and ultimately higher morbidity and mortality in stroke units. Subsequent investigations in both animal models and clinical populations demonstrated that the neuroprotective or deleterious effects of T-cells are not determined by the overall lymphocyte count, but rather by the relative abundance and functional characteristics of specific T-cell subtypes, as illustrated in Fig. 3 (Gu et al., 2015).

T-lymphocytes exert multifaceted effects in acute ischemic stroke, with their impact largely determined by subtype-specific functions. Multiple T-cell subsets become activated through interactions with innate immune cells, including macrophages and phagocytes, thereby sustaining a prolonged subacute phase of neuroinflammation (Cao et al., 2023)**.** In later stages, T-cell infiltration may further increase as a consequence of blood–brain barrier (BBB) disruption. BBB breakdown, together with the release of antigens from necrotic tissue, facilitates the entry and accumulation of autoreactive B- and T-lymphocytes within the central nervous system.

The functional heterogeneity of T-cells results in divergent effects on stroke outcomes. Proinflammatory CD4^+^ T-cell subsets—most notably Th1 and Th17 cells—have been consistently associated with increased tissue injury and poorer clinical prognosis. Th1 cells secrete cytokines such as interleukin-2 (IL-2), IL-12, interferon-γ (IFN-γ), and tumor necrosis factor (TNF), all of which contribute to further BBB compromise. In murine stroke models, antigen recognition by Th1 cells promotes elevations of IFN-γ and transforming growth factor-β (TGF-β) within the brain parenchyma, correlating with worsened neurological outcomes. Th17 cells amplify the cytotoxic activity of CD8^+^ T-cells and enhance neutrophil recruitment and activation in ischemic tissue, thereby promoting infarct expansion. Additionally, the presence of autoreactive B and T cells in the post-stroke inflammatory milieu has been linked to an increased susceptibility to secondary infections—particularly respiratory—contributing to increased morbidity (Malone et al., 2019)**.** Consequently, immunomodulatory strategies aimed at limiting T-cell infiltration into ischemic brain regions are currently under investigation as potential therapeutic approaches (Malone et al., 2019)**.**

Among the T-cell subpopulations implicated in cerebrovascular pathology, CD4^+^CD28^−^ T cells represent a particularly interesting group. CD28, a key co-stimulatory molecule, is essential for T-cell activation, differentiation, and survival. Altered CD28 expression leads to the emergence of distinct T-cell subsets with different immunological properties, some of which have been associated with chronic inflammation and vascular injury. Growing evidence suggests that CD28-related immune mechanisms contribute equally to the development as well as the progression of ischemic stroke, influencing not only vascular integrity but post-stroke neuroinflammatory responses as well (Nowik et al., 2007)**.**

CD4^+^CD28^−^ T lymphocytes—often referred to as CD28-null cells—constitute a population of highly differentiated, cytotoxic, and pro-inflammatory T cells. In the setting of acute ischemic stroke, multiple studies have demonstrated significantly elevated circulating levels of CD4^+^ and CD4^+^CD28^−^ T cells in affected patients compared with non-stroke controls. Moreover, the frequency of CD28-null cells appears to vary according to TOAST stroke subtype. Notably, patients with cardioembolic stroke exhibit the highest proportions of CD28-null T cells. Their circulating levels also correlate positively with clinical severity as assessed by the National Institutes of Health Stroke Scale (NIHSS) and the Scandinavian Stroke Scale (SSS). Receiver operating characteristic (ROC) analyses further suggest that CD28-null T-cell percentages may hold diagnostic value in distinguishing between ischemic stroke subtypes. Collectively, these findings support the role of CD4^+^CD28^−^ lymphocytes as biomarkers of immune activation and disease severity in acute ischemic stroke (Tuttolomondo et al., 2015).

The role of CD4^+^CD28^−^ lymphocytes extends even to the presence of atherosclerotic disease, a major etiological contributor to ischemic stroke. In patients undergoing carotid endarterectomy, increased circulating proportions of CD28-null lymphocytes have been associated with plaques characterized by abundant collagen fibers and cholesterol crystals but limited calcification—features indicative of an unstable plaque phenotype. The cytotoxic properties of these lymphocytes may promote plaque destabilization and rupture, thereby facilitating thromboembolic events as cause of acute cerebral ischemia (Masztalewicz et al., 2013).

Furthermore, elevated levels of CD28-null lymphocytes have also been found in individuals without a history of previous cerebral ischemia but with established cardiovascular risk factors such as arterial hypertension or diabetes mellitus, suggestive of a preceding role of this T-cell subset in cerebrovascular events. Additionally, these findings underpin a possible role CD28-null cells in actively promoting vascular inflammation and thrombogenesis, rather than representing a secondary consequence of ischemic injury.

While the loss of CD28 expression on effector T cells is associated with heightened vascular inflammation, activation of CD28 signaling can exert protective, anti-inflammatory effects through the expansion of regulatory T cells (Tregs). CD28-stimulated Tregs demonstrate enhanced functional activity, including increased proliferation and elevated secretion of interleukin-10 (IL-10), a key immunomodulatory cytokine. This regulatory response attenuates post-stroke neuroinflammation and contributes to tissue protection, indicating that CD28-mediated Treg augmentation may represent a promising therapeutic strategy to mitigate ischemic brain injury.

Taken together, these findings highlight a dual immunological role for CD28 in ischemic stroke. Loss of CD28 expression identifies cytotoxic, pro-inflammatory T-cell subsets that promote vascular inflammation, plaque destabilization, and increased susceptibility to ischemic events. In contrast, CD28 co-stimulation drives the expansion of Tregs that suppress inflammatory responses and support tissue repair following cerebral ischemia. The interplay between these opposing CD28-dependent pathways may be crucial for stroke onset, evolution of ischemic injury and recovery (Na et al., 2015)**.**

Another T-cell subgroup exhibiting neuroprotective properties are CD4^+^ Th2 cells. They show anti-inflammatory effects through secretion of cytokines, such as IL-4, IL-5, IL-10, and IL-13, which promote tissue repair and subsequent neurological recovery (Arumugam et al., 2005)**.** These cells contribute further to the reduction of post-stroke infections, a major risk factor leading to increased morbidity and mortality (Arumugam et al., 2005)**.** In conjunction with regulatory T cells (Tregs), which secrete nerve growth factor (NGF), Th2 cells facilitate angiogenesis, clearance of cellular debris, and tissue repair (Cao et al., 2023; Malone et al., 2019)**.**

Treg populations, initially sparse in healthy cerebral tissue, increase significantly within the infarcted area shortly after an ischemic event. Their activation is mainly driven by IL-2, IL-33, and serotonin-mediated stimulation of T-cell receptors, while chemokines such as CCL1 and CCL20 promote tissue-infiltration. Tregs confer neuroprotection via multiple mechanisms: they promote oligodendrocyte regeneration, inhibit neurotoxic astrogliosis through secretion of amphiregulin, and facilitate white matter repair (Ito et al., 2019a)**.** Furthermore, several studies have demonstrated that Tregs effectively suppress neuroinflammation within ischemic lesions, thereby limiting secondary tissue injury (Liesz et al., 2015)**.**

Beyond suppressing inflammation, Tregs actively support microglial regeneration by modulating gene expression pathways that enhance reparative processes. This regenerative function is closely associated with IL-10 production, resulting in reduced infarct volume and improved tissue recovery (Benakis et al., 2022)**.**

These mechanistic insights have inspired novel immunotherapeutic strategies utilizing T-cell therapy. Recent studies have explored the transplantation of bone marrow–derived stem cells (BMSCs) following ischemic stroke, with Tregs within BMSCs acting as key mediators of immunomodulation and neuroprotection. This approach has been shown to enhance neuronal recovery, improve oxygen delivery, and support glucose metabolism—factors expecting to improve the survival of vulnerable neuronal and glial populations (Neal et al., 2019a). In addition to elevated IL-10 levels, production of IL-6 has been shown to be increased, further promoting recovery and repair of neurons and microglia, which are highly susceptible to hypoxic injury.

However, although Tregs may limit a maladaptive inflammatory process, they may also contribute to microvascular dysregulation (Xu et al., 2013)**.** Interestingly, clinical models indicate potential sex-specific differences in T-cell populations and function following stroke, which may influence prognosis and long-term recovery (Yan et al., 2012)**.**

Immunoregulatory strategies targeting Treg cells—such as monoclonal antibodies and cytokine modulators—aim to suppress proinflammatory pathways (e.g., TNF-α, IL-1) while enhancing anti-inflammatory mediators like IL-6 and IL-10. While T-cell subgroups, such as Th1-cells, produce reactive oxygen species (ROS) and proinflammatory cytokines, Th2-cells secrete anti-inflammatory factors including IL-4 and IL-10, which stimulate NGF production, tissue repair, and clearance of cellular debris (Heindl et al., 2021)**.**

#### 3.1.3 Natural killer cells

Natural killer (NK) cells play a critical role in the immune response following cerebral ischemia by modulating immune activation and contributing to post-ischemic neuroinflammation. After an ischemic stroke, NK cells infiltrate the affected brain regions and exacerbate neuronal injury through the release of interferon-γ (IFN-γ). They also promote disruption of the blood–brain barrier (BBB) via the chemokine IP-10 (CXCL10) and its receptor CXCR3, thereby amplifying the local inflammatory cascade and increasing neuronal hyperexcitability—processes that collectively contribute to expansion of the ischemic lesion volume (Zhang et al., 2014)**.** In addition to these pro-inflammatory functions, NK cells participate in the development of post-stroke immunosuppression, a well-recognized phenomenon associated with heightened susceptibility to systemic infections and poorer clinical outcomes (Gan et al., 2014)**.**

Cerebral ischemia further influences NK-cell biology by impairing their functional capacity through alterations in neurogenic and intracellular signaling pathways, including mechanisms involving post-transcriptional regulation (Chen et al., 2019)**.** Recent evidence has identified microRNA-1224 as a potential negative regulator of NK-cell activation, suggesting a novel therapeutic target for modulating neuroimmune interactions and reducing infection risk in the post-stroke period (Feng et al., 2021)**.**

### 3.2 Medical interventions based on clinical and experimental studies

A broad range of pharmacological agents has been investigated for their potential to modulate neuroinflammation following acute ischemic stroke, aiming to improve neurological outcomes. Multiple therapeutic strategies have been developed to attenuate post-stroke neuroinflammatory processes and enhance neuroprotection. In this context, we reviewed the literature focusing on clinical studies that evaluated the efficacy of various agents—including statins, antiplatelet and anticoagulant drugs, novel pharmacological compounds, monoclonal antibodies, vitamins, and herbal products—used in patients with ischemic stroke to limit neuroinflammation and promote recovery.

**Heparin**, due to its potent anticoagulant properties, has been used during the acute phase of ischemic stroke. Clinical studies suggest that heparin administration is associated with reduced morbidity and mortality in stroke patients compared to placebo, and similar benefits have been observed in patients with severe sepsis (Chamorro et al., 2000; Levi et al., 2007). Notably, heparin use was not associated with a significant increase in hemorrhagic complications, and its protective effects extended to populations with severe cardiovascular comorbidities, demonstrating a reduction in stroke incidence and improved safety profile (Levy et al., 2009).

**Antiplatelet agents** remain a cornerstone of acute and long-term management of ischemic stroke, serving both to improve clinical outcomes and prevent recurrence. Recent evidence indicates that the combination of aspirin with extended-release dipyridamole may offer additional benefit in the early phase of stroke by attenuating neuroinflammatory pathways, thus contributing to better clinical recovery in selected patients (Worthmann et al., 2012a). Furthermore, triflusal, a 4-fluoromethyl derivative of salicylic acid, has demonstrated anti-inflammatory effects by reducing levels of pro-inflammatory cytokine IL-6 and chemokines such as MIP-1 and MCP-1, both implicated in promoting necrotic damage in the ischemic brain (Alvarez-Sabín et al., 2009).

**Statins** represent an important component in the management of ischemic stroke due to their lipid-lowering and pleiotropic effects. Primarily, statins reduce low-density lipoprotein (LDL) cholesterol levels, thereby limiting the progression of atherosclerosis—a major risk factor for ischemic stroke, myocardial infarction, and thrombotic vascular events. Elevated LDL cholesterol is not only atherogenic but also contributes to systemic inflammation by stimulating the production of pro-inflammatory cytokines and interleukins (Montaner et al., 2008a)**.**

Recent clinical studies have demonstrated that combination therapy with statins and ezetimibe significantly reduces the volume of inflamed vascular lesions and suppresses thrombin generation and activity, ultimately improving clinical outcomes (van Kuilenburg et al., 2011a). Moreover, specific statins exhibit distinct anti-inflammatory effects: pravastatin has been shown to lower circulating C-reactive protein (CRP), while atorvastatin reduces levels of IL-6 and TNF-α, biomarkers associated with poor stroke prognosis (Kitagawa et al., 2017a; Yu et al., 2017a). Animal studies have further supported the neuroprotective effects of statins, demonstrating dose-dependent reductions in neuronal injury and neuroinflammation (REF).

**Paracetamol**, though not a direct anti-inflammatory agent, has been shown to confer benefit in the acute stroke setting by reducing fever, which is associated with worse functional outcomes post-stroke. Several studies have reported improved morbidity and mortality rates in patients treated with paracetamol compared to placebo during the acute phase of stroke (de Ridder et al., 2015a; Ridker et al., 1997a).

Conversely, **colchicine**, an anti-inflammatory agent used in other vascular and inflammatory conditions, has not demonstrated significant benefit in ischemic stroke. Clinical data indicate no improvement in inflammatory markers such as CRP or platelet activity, and outcomes were comparable to those observed in placebo-treated patients (Raju et al., 2012a, 2012b).

**Interleukin-10 (IL-10)**, a potent anti-inflammatory cytokine, has shown promise in modulating post-stroke inflammation, improving outcomes in both ischemic stroke and intracerebral hemorrhage models (Pelidou et al., 1999). **Vinpocetine**, a synthetic derivative of the vinca alkaloid, exerts immunomodulatory effects by inhibiting the kappa light chain of B-cell activation, thereby supporting neural recovery in post-stroke patients (Zhang et al., 2018).

A combination of anti-inflammatory and antioxidant therapies may further enhance neuroprotection by reducing ROS, which are implicated in ischemia-induced neuronal damage. Agents such as **mitoquinone**, a mitochondria-targeted antioxidant, and **lubeluzole**, a nitric oxide modulator, have demonstrated protective effects by mitigating oxidative stress and necrosis. Other ROS scavengers, including **edaravone** and **ebselen**, have also shown efficacy in reducing oxidative damage following stroke (Bi and Song, 2011; Shirley et al., 2014). Additionally, a compound blend of plant- and animal-derived enzymes—**flogenism**, containing bromelain, trypsin, and rutin—has been associated with anti-inflammatory, fibrinolytic, and antithrombotic activity, though further large-scale studies are needed to validate these findings (Vinychuk and Cheren'ko, 2007).

In contrast however, **NXY-059**, a free radical-trapping agent administered within 6 h of stroke onset, failed to demonstrate clinical benefit in randomized controlled trials, highlighting the ongoing challenges in translating neuroprotective strategies into effective stroke therapies (Diener et al., 2008).

Recent research has explored the potential application of immunomodulatory agents—commonly used in autoimmune conditions such as multiple sclerosis—for mitigating neuroinflammation following acute ischemic stroke. Several clinical studies have evaluated the combination of **alteplase** with immunomodulators such as **fingolimod**, demonstrating improved clinical outcomes and a significant reduction in post-stroke neuroinflammation (Raju et al., 2012a, 2012b). Notably, these studies also reported enhanced rates of vascular recanalization and a marked decrease in reperfusion injury (Zhang et al., 2017; Zhu et al., 2015; Bo et al., 2024).

**Dimethyl fumarate (DMF)** has also emerged as a promising therapeutic candidate due to its dual immunomodulatory and antioxidant effects. Preclinical studies have shown that DMF significantly reduces infiltration of immune cells, including neutrophils, macrophages, and dendritic cells, while simultaneously increasing the presence of neurons and activated microglia in the peri-infarct zone, leading to reduced infarct volume and improved histological outcomes (Owjfard et al., 2023; Zhang et al., 2024).

**Interferon beta-1a**, widely used in multiple sclerosis, exerts anti-inflammatory effects by suppressing pro-inflammatory cytokine production and preserving blood-brain barrier integrity. This mechanism may have a potent neuroprotective role limiting neuronal injury and has been associated with reduced morbidity and mortality following ischemic stroke (Kuo et al., 2016; Wanve et al., 2019)**.**

Evidence regarding **cell-based therapies** and immunotherapeutic approaches using monoclonal antibodies in ischemic stroke remains limited. To date, lymphocyte-based cell therapy is neither well understood nor widely implemented in stroke management. Nevertheless, preliminary investigations have yielded promising findings. Effective immunomodulatory therapy for ischemic stroke requires a precise understanding of the mechanisms governing T- and B-lymphocyte activation, function, and interaction within the neuroimmune environment. Emerging data suggest that enhancement of CD8^+^ regulatory T cells (CD8^+^ Tregs) may suppress inflammatory responses through induction of interleukin-10 (IL-10) and promote neuronal repair via epidermal growth factor–like transforming growth factor (ETGF) signaling, thereby contributing to long-term neurological recovery (Cai et al., 2022)**.**

Recent experimental studies also support the therapeutic potential of bone marrow–derived stem cell transplantation. These cells may function as active immunomodulatory mediators, exerting neuroprotective effects, enhancing oxygen and glucose delivery, and facilitating earlier recovery by shielding neurons from ischemia-induced cytotoxic stimuli (Neal et al., 2019b)**.**

Monoclonal antibodies have also been explored as potential modulators of post-stroke inflammation. Enlimomab and natalizumab—agents targeting leukocyte adhesion and CNS leukocyte migration, respectively—were evaluated for their ability to attenuate neuroinflammation. However, clinical trials failed to demonstrate therapeutic benefit in stroke populations; in some instances, treatment was associated with worsened outcomes, potentially due to off-target immunological effects or compromised systemic immune surveillance (Enlimomab Acute Stroke Trial Investigators, 2001; Simats et al., 2016; Elkind et al., 2020a)**.**

Beyond direct immune-modulators, several pharmacologic agents used for managing chronic comorbidities such as hypertension and diabetes mellitus have demonstrated secondary neuroprotective properties. **Angiotensin-converting enzyme inhibitors (ACEIs)** and **angiotensin receptor blockers (ARBs)**, by inhibiting the renin–angiotensin system (RAS), may confer anti-inflammatory and antithrombotic benefits. Evidence suggests RAS contributes not only to blood pressure regulation but also to vascular inflammation and platelet activation. Thus, RAS inhibition may reduce the risk of thrombotic events, including myocardial and cerebral infarction (Chae et al., 2014).

In addition, **pioglitazone**, a thiazolidinedione, has shown promise in the post-ischemic period by attenuating neuronal injury and limiting subsequent neurodegeneration (Culman et al., 2012)**.** Similarly, **glucagon-like peptide-1 receptor agonists (GLP-1RAs)** have demonstrated significant antioxidant and anti-inflammatory properties in experimental models. These agents reduce infarct volume, suppress apoptosis, promote neurogenesis, and enhance cerebral blood flow, even in normoglycemic stroke patients (Goldenberg et al., 2022). Lastly, **allopurinol**, traditionally used in hyperuricemia management, may also exert neuroprotective effects by inhibiting the expression of intercellular adhesion molecule-1 (ICAM-1), thereby suppressing leukocyte-mediated inflammation and improving clinical outcomes following stroke (Muir et al., 2008).

Furthermore, a growing body of research is exploring the neuroprotective potential of various pharmacologic and non-pharmacologic interventions in the treatment of acute ischemic stroke, though further clinical validation is necessary. **DL-3-n-butylphthalide (NBP)**, a compound derived from celery seed oil, has demonstrated significant neuroprotective effects. Clinical trials have reported improved functional outcomes at 90 days post-stroke in patients treated with NBP compared to placebo (Lv et al., 2022). Similarly, **mildronate**, an inhibitor of carnitine-dependent fatty acid oxidation, has shown promise as a safe and potentially effective agent in acute carotid and cerebral infarctions. Its mechanism includes enhancement of cerebral blood flow and reduction of ischemic injury (Zhu et al., 2013).

**Sodium valproate**, commonly used as an antiepileptic drug, also functions as a histone deacetylase 9 (HDAC9) inhibitor. Given HDAC9's established association with large-artery stroke, valproate may exert neuroprotective effects by preserving neuronal synaptic integrity and reducing recurrent stroke risk. Ongoing research is evaluating its role in post-stroke neuroplasticity and secondary prevention (Brookes et al., 2018).

Vitamins—particularly **vitamin C** and **B-complex vitamins**—have garnered interest for their **antioxidant** and **anti-inflammatory** properties. Low serum levels of these water-soluble vitamins have been associated with increased oxidative stress, elevated inflammatory markers (e.g., CRP, MCP-1, and homocysteine), and poorer outcomes following ischemic stroke (Sánchez-Moreno et al., 2004; Ullegaddi et al., 2006). Supplementation with B-complex vitamins may promote atherosclerotic plaque stability, potentially reducing the risk of recurrent vascular events by modulating inflammation at the level of the vascular intima and media tunica of the carotid arteries (Nenseter et al., 2009; Potter et al., 2009).

In addition to conventional agents, **traditional Chinese medicine (TCM)** products have been studied for their neuroprotective potential. **BNG-1**, a compound mixture with antithrombotic properties comparable to aspirin, has shown preliminary promising results, though its efficacy as a thrombolytic agent remains under investigation (Chang et al., 2015). **Licorice** has also been reported to contribute to neurological improvement in AIS patients (Ravanfar et al.).

Several natural compounds have demonstrated anti-inflammatory and neuroprotective effects. **Ginsenoside Rd**, derived from *Panax ginseng*, has been shown to suppress microglial proteasome activity with fewer adverse effects than glucocorticoids, contributing to reduced neuroinflammation (Zhang et al., 2016). **Ginkgolide** and **melatonin** have also been associated with improved functional and neurological outcomes post-stroke. These benefits were associated with improved NIHSS scores in clinical trials (Dong et al., 2020; Mehrpooya et al., 2022). Lastly, **acupuncture** has been studied as an adjunctive therapy. Evidence suggests that it may improve neurological function and attenuate inflammatory responses, potentially supporting neural recovery in stroke patients (Tsai et al., 2024).

## 4 Discussion

Ischemic stroke can have devastating consequences for affected individuals. The reduction in cerebral blood flow leads to neuronal death directly through severe ischemia and indirectly via mechanisms such as glutamate excitotoxicity, oxidative stress, calcium dysregulation, and inflammation (Moskowitz et al., 2010). The inflammatory response to focal ischemia of brain tissue begins within hours of stroke onset and may persist for weeks (Li et al., 2021), contributing significantly to the expansion of the ischemic core and influencing patient clinical outcome.

The immune response following ischemic stroke involves both the innate and adaptive immune systems. The initial activation of innate immunity is rapid and non-specific, enabling the recognition of danger-associated molecular patterns (DAMPs) and triggering a broad immune reaction involving neutrophils, monocytes, macrophages, and microglia (Iadecola et al., 2020). While lymphocytes may be activated early, adaptive immunity becomes prominent days after the stroke and involves antigen-specific responses via immunoglobulins and T-cell receptors (Jain et al., 2017).

**Fig. 4:**
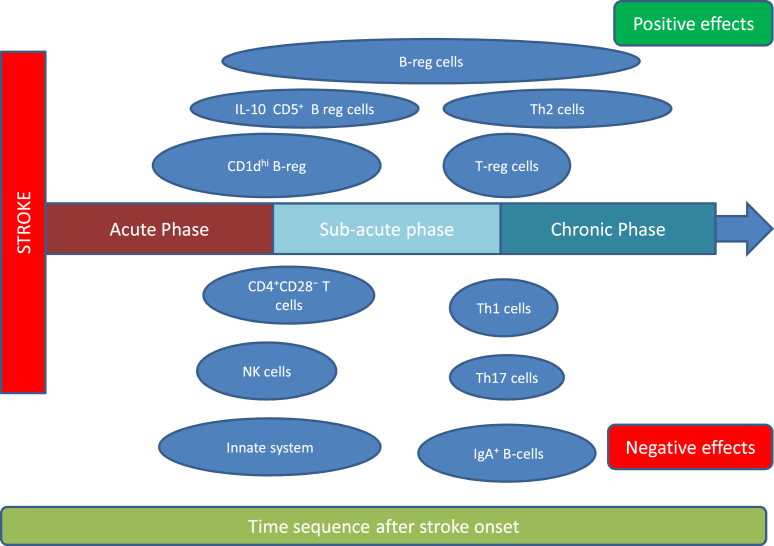
Proposed temporal sequence of activation and duration of action of variable cell subgroups in post stroke neuroinflammation.

Following ischemic injury, brain-derived antigens are released and presented by antigen-presenting cells, leading to B- and T-cell activation and expansion in the spleen and lymph nodes. Moreover, cells of the innate immunity phagocytose dead cells in the ischemic brain tissue leading to production of antigen presenting cells, which consequently induce the expansion of B- and T-cells as we can see to Fig. 4 (Shichita et al., 2009). Macrophages expressing MHC class II receptors near T-cell zones in the tonsils and cervical lymph nodes of stroke patients have been shown to present both neuronal- and myelin-derived antigens (Planas et al., 2012). Tregs may accumulate in the brain during the chronic recovery phase, independent of innate immune activity. These T-cells express genes associated with neural processes and contribute to neuroprotection by suppressing neurotoxic astrogliosis, while brain infiltration and amplification of T-cells depends on interleucins and chemokines (Ito et al., 2019b). B-cells may also infiltrate the brain post-stroke, enhancing Treg activity and playing a neuroprotective role (Bodhankar et al., 2013b; Ren et al., 2011).

Experimental studies indicate that B-cells suppress activation of both T-cells and innate immune cells through IL-10 secretion, reducing inflammation and infarct volume while improving functional outcomes. Additionally, a significant increase of Treg cells was noted, leading to a reduced proinflammatory milieu. These findings were related with reduced infarct volume and better outcomes overall. IL-10 also exerts a direct neuroprotective effect on cortical neurons and may alter neuronal susceptibility to excitotoxic damage (Sharma et al., 2011; Grilli et al., 2000).

Beyond immune regulation, the adaptive immune system contributes to tissue repair and recovery by reducing infarct size and facilitating perfusion (Wu et al., 2024b). B-cells, through mechanisms such as ischemic preconditioning, may protect against future stroke events by reprogramming cellular responses to injury (Gesuete et al., 2016c). Moreover, IL-10 promotes neurotrophin production in the infarct area (de Bilbao et al., 2009), and B-cells may exert neurotrophic effects even in the absence of IL-10, as demonstrated in vitro (Ortega et al., 2020; Malone et al., 2023).

Tregs are also crucial for post-stroke recovery. They mitigate white matter damage (Shi et al., 2021)**,** suppress excessive astrogliosis (Ito et al., 2019b), and support neurogenesis, cell migration and oligodendrogenesis during late recovery stages (Wang et al., 2015). However, not all lymphocyte-mediated responses are beneficial. IgA + B-cells have been implicated in post-stroke cognitive decline, with anti-CD20-mediated B-cell depletion reducing cognitive deficits in experimental models suggesting a negative role of B-lymphocytes in delayed cognitive impairment (Bernardo-Castro et al., 2020). Similarly, Th17-cells may exacerbate stroke injury by promoting inflammation, enhancing blood–brain barrier permeability, facilitating peripheral immune cell infiltration, worsening neuronal cell death and inhibiting neurogenesis, all of which contribute to cognitive dysfunction (Wang et al., 2024). Natural killer cells have also been shown to negatively influence stroke outcomes, as mentioned above.

This dual role of the immune system in both injury and recovery has prompted investigation into immunomodulatory therapies for ischemic stroke. Many medications used for secondary stroke prevention—such as antiplatelets and statins—exhibit additional anti-inflammatory effects in the ischemic brain (Worthmann et al., 2012b; Alvarez-Sabin et al., 2009; Montaner et al., 2008b; van Kuilenburg et al., 2011b; Kitagawa et al., 2017b; Yu et al., 2017b; de Ridder et al., 2015b; Ridker et al., 1997b). Various experimental agents with anti-inflammatory or antioxidant properties have shown promise in preclinical models, though clinical data remains limited.

Of particular interest are monoclonal antibodies such as enlimomab and natalizumab, which demonstrated disappointing results in clinical trials (Enlimomab Acute Stroke Trial, 2001; Elkind et al., 2020b). Enlimomab, which targets neutrophil adhesion molecule, was associated with worsened outcomes and increased mortality, underscoring the complex role of inflammation in tissue repair. The failure of natalizumab—a known potent CNS inflammation modulator—has been extensively debated. Factors such as treatment timing, elder patient comorbidities, stroke type and conventional stroke risk factors, as well as publication bias may contribute to the observed lack of efficacy (Mizuma et al., 2017). Preclinical studies suggest that VLA-4 antibody treatments may be beneficial only in small infarcts, further supporting the notion that stroke subtype can influence immune responses and therapeutic efficacy (Llovera et al., 2015).

Safety concerns also complicate the use of immunomodulatory therapies in the acute stroke setting. For example, Canakinumab, an IL-1β monoclonal antibody, reduced cardiovascular events in patients with previous myocardial infarction in the CANTOS (Canakinumab Antiinflammatory Thrombosis Outcome Study) trial, but also increased fatal sepsis risk (Ridker et al., 2017). Furthermore, stroke-induced immunosuppression—characterized by decreased lymphocyte counts and impaired host defense in the acute phase in stroke patients —predisposes patients to infections such as stroke-associated pneumonia. Additional immunosuppressive or immunomodulating interventions, although potentially beneficial for limiting brain inflammation, may exacerbate infection risk and worsen overall prognosis (Faura et al., 2021).

In summary, according to current evidence, immune system activation following ischemic brain injury evolves along two interrelated axes and undergoes continuous modulation beginning in the acute ischemic phase and continuing to subacute and ultimately chronic phase. On one axis, this process involves the activation and relative proportions of innate and adaptive immune cells, as well as the circulating levels of interleukins and other inflammatory mediators. On the other axis, the functional role and impact of specific immune cell subpopulations dynamically change over time. The influence of different lymphocyte subsets may therefore be determined not only by their cellular type but also by the timing of activation and the local context within the evolving brain injury. Effective therapeutic modulation of these immunological pathways requires a comprehensive understanding of both the temporal progression of post-ischemic inflammatory responses and the microenvironmental milieu in which lymphocyte activation occurs, as these factors collectively shape the balance between neuroprotection and tissue injury (Xu et al., 2021)**.**

As extensively reviewed here, numerous pharmacological agents have been investigated for the treatment of ischemic stroke and modulation of post-stroke neuroinflammation, yet overall clinical outcomes remain limited. Some of the beneficial effects of commonly used medications, such as statins or antihypertensive agents, have been partially attributed to their anti-inflammatory properties. Other substances have also shown potential positive effects. However, the failure of targeted immunomodulatory therapies to meaningfully alter stroke progression highlights the current gaps in our understanding of the underlying immunological pathways.

In the acute phase of ischemic injury, the immediate necrosis within the infarct core triggers rapid activation of the innate immune system and disruption of the blood–brain barrier, which leads eventually to the activation of adaptive immune system. Activation of the adaptive immune system seems to occur typically during the subacute phase and is often associated with a proinflammatory, potentially detrimental response. In contrast, activation of regulatory B and T cells, which exert neuroprotective effects and improve stroke prognosis, generally arises in later subacute or during the chronic phase and may persist over time. This temporal heterogeneity may explain the limited efficacy of non-specific, undifferentiated immunotherapies (Ran et al., 2020)**.** Targeted strategies that selectively suppress lymphocyte subtypes with detrimental effects, while promoting or even temporally shifting the activation and CNS migration of protective subsets, hold potential to favorably modify the trajectory of ischemic brain injury, while limiting the adverse reactions. The efficacy of targeted immunomodulatory interventions is likely influenced by the specific lymphocyte subpopulation addressed, the timing and duration of treatment, and possibly related to the underlying stroke subtype. These factors should be carefully considered in the design of future studies. Importantly, modulation of post-stroke neuroinflammation, when implemented alongside current revascularization therapies, holds the potential to achieve a significant advance in the treatment of ischemic stroke. Therefore, further research is warranted to shed light the spatial and temporal evolution of post-stroke neuroinflammation, which is essential for guiding precise and effective immunomodulatory interventions.

## 5 Conclusions

Lymphocytes play a pivotal and emerging role in the pathophysiology of acute ischemic stroke, significantly influencing the progression of post-stroke inflammation. Therefore, they seem to be a novel therapeutic target in post stroke patients. This review summarized current evidence on the functions of B- and T-lymphocytes during both the early and late stages of stroke. The data indicate systemic immune activation characterized by a regulated sequence of anti-inflammatory cytokine release and protective mechanisms. However, certain immune responses may contribute to adverse outcomes such as cognitive impairment and dementia.

We also discussed the anti-inflammatory properties of commonly used stroke medications and other therapeutic substances. Despite promising findings, data from human subjects remain limited, and translating results from animal models to clinical practice is challenging, as exemplified by the failure of natalizumab. Additionally, safety concerns related to immune modulation in stroke patients are not yet fully understood.

Future research should aim to further clarify the specific roles of lymphocyte subpopulations in post-stroke inflammation and tissue repair, as well as their temporal evolution. This will enable the development of targeted therapies that mitigate detrimental inflammatory effects while preserving peripheral immune competence to avoid increasing infection risks. Moreover, enhancing the neuroprotective and reparative functions of the immune system within the CNS, along with optimizing the timing and duration of such treatments, remain critical and yet unresolved challenges.

## CRediT authorship contribution statement

**A. Papageorgiou:** Methodology, Investigation, Formal analysis, Conceptualization. **D. Chatzistefanidis:** Supervision, Methodology, Formal analysis, Data curation. **M. Nikolakea:** Writing – original draft, Software. **N.-R. Karela:** Writing – review & editing, Formal analysis. **M. Gkrampovari:** Software, Methodology, Formal analysis. **K. Lantavos:** Writing – original draft, Methodology. **D. Bartzi:** Writing – review & editing, Writing – original draft. **L. Traikov:** Visualization, Supervision. **S. Markoula:** Visualization, Supervision.

## Declaration of Competing Interest

The authors declare there are no conflicts of interst as well as no financial support by any of the authors.

## Data Availability

No data was used for the research described in the article.
